# A founder mutation in the *PLPBP* gene in families from Saguenay‐Lac‐St‐Jean region affected by a pyridoxine‐dependent epilepsy

**DOI:** 10.1002/jmd2.12196

**Published:** 2021-02-23

**Authors:** Maitou Pal, Baiba Lace, Yvan Labrie, Nathalie Laflamme, Nadie Rioux, Samarth Thonta Setty, Marc‐Andre Dugas, Louise Gosselin, Arnaud Droit, Nicolas Chrestian, Serge Rivest

**Affiliations:** ^1^ Faculty of Medicine Laval University Québec Québec Canada; ^2^ Department of Medical Genetics Centre Mère Enfant Soleil, Laval University Québec Québec Canada; ^3^ Centre de recherche CHU de Québec‐ Université Laval, Laval University Québec Québec Canada; ^4^ Department of Pediatrics Centre Mère Enfant Soleil, Laval University Québec Québec Canada; ^5^ Department of Pediatric Neurology, Pediatric Neuromuscular Disorder Centre Mère Enfant Soleil, Laval University Québec Québec Canada

**Keywords:** PLPBP, PROSC, vitamin B6‐dependent early‐onset epilepsy

## Abstract

Pyridoxine‐dependent epilepsy (PDE) is a relatively rare subgroup of epileptic disorders. They generally present in infancy as an early onset epileptic encephalopathy or seizures, refractory to standard treatments, with rapid and variable responses to vitamin B6 treatment. Whole exome sequencing of three unrelated families identified homozygous pathogenic mutation c.370_373del, p.Asp124fs in *PLPBP* gene in five persons. Haplotype analysis showed a single shared profile for the affected persons and their parents, leading to a hypothesis about founder effect of the mutation in Saguenay‐Lac‐St‐Jean region of French Canadians. All affected probands also shared one single mitochondrial haplotype T2b3 and two rare variations in the mitochondrial genome m.801A>G and m.5166A>G suggesting that a single individual female introduced *PLPBP* mutation c.370_373del, p.Asp124fs in Quebec. The mutation p.Asp124fs causes a severe disease phenotype with delayed myelination and cortical/subcortical brain atrophy. The most noteworthy radiological finding in this Quebec founder mutation is the presence of the temporal cysts that can be used as a marker of the disease. Also, both patients, who are alive, had a history of prenatal supplements taken by their mothers as antiemetic medication with high doses of pyridoxine. In the context of suspected PDE in patients with neonatal refractory seizures, treatment with pyridoxine and/or Pyridoxal‐5‐phophate has to be started immediately and continued until the results of genetic analysis received. Even with early appropriate treatment, neurological outcome of our patient is still poor.

**Synopsis**

We identified a founder mutation in French Canadian population that will help to rapidly identify and provide better prenatal diagnosis in this population.

## 1 INTRODUCTION

Pyridoxine‐dependent epilepsy (PDE) is a rare subgroup of epileptic disorders. They generally present in infancy as an early onset epileptic encephalopathy, refractory to standard treatments, with rapid response to vitamin B6 treatment.

The first reported case dates back to 1954 in an infant with refractory seizures in whom a positive response was observed after a treatment comprising of many vitamins, including B6.^1^

PDE is associated with a range of different presentations, but typically it is characterized by a variety of seizure types. The onset of the disease can range from neonatal to early infancy, but an onset of up to 3 years has been reported. They are generally unresponsive to standard anticonvulsants but have a fast response to the administration of pyridoxine and remain dependent on it and any interruptions lead to recurrence.^2^

**FIGURE 1:**
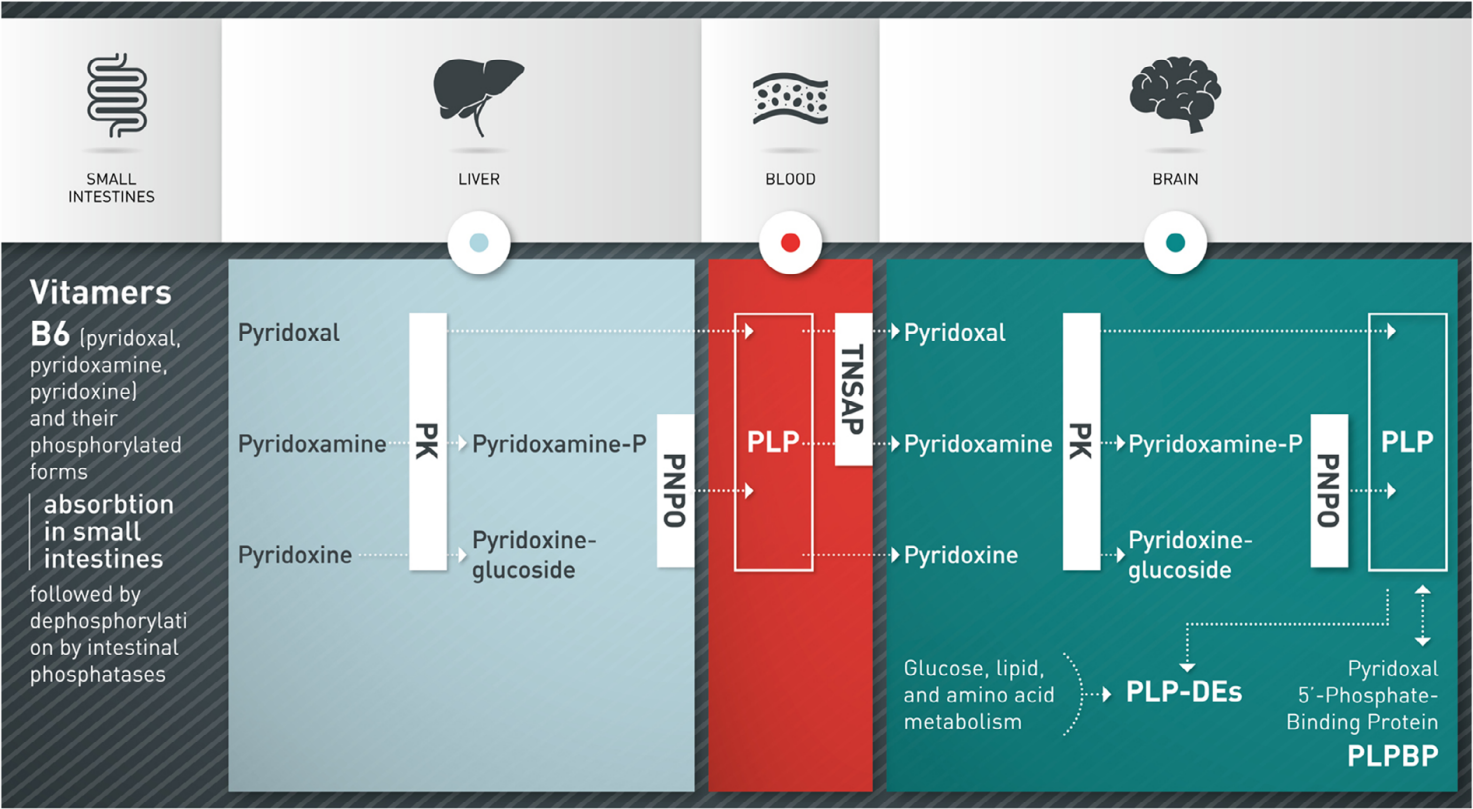
Pyridoxine metabolism. IP, intestinal phosphatases; PK, pyridoxine kinase; PLP, Des PLP dependent enzymes; PLP, pyridoxal 5′‐phosphate. PLPBP binds PLP and might participate in storage of PLP

Pyridoxine is a water‐soluble vitamin that can be found in a variety of food sources. There are three vitamin B6 vitamers absorbed in the small intestine: pyridoxal, pyridoxamine, pyridoxine, and their phosphorylated forms (Figure 1). In order to allow cellular uptake and to cross the blood‐brain barrier, the phosphorylated forms must be dephosphorylated by intestinal phosphatases and tissue nonspecific alkaline phosphatase (TNSALP). Once within the cell, these vitamers are rephosphorylated and oxidized resulting in the formation of pyridoxal 5′‐phosphate (PLP) by kinases and pyridox‐(am) ine 5′‐phosphate oxidase (PNPO), respectively. PLP is a cofactor in humans, involved in over 140 enzymatic reactions, including neurotransmitter synthesis and degradation.^3^

Several disorders have been identified as causes of PDE: antiquitin deficiency, PNPO deficiency, hypophosphatasia, PLPBP deficiency, and hyperprolinemia type 2. A couple other disorders may show certain response to B6 treatment like molybdenum cofactor deficiency and glycosylphosphatidylinositol biosynthesis defects.^4^ Antiquitin deficiency was previously shown to be associated with PDE via Knoevenagel condensation reaction by inactivation of PLP.^5^ Hyperprolinemia type II is associated with deficient activity of delta‐pyroline 5‐carboxylate (P5C) dehydrogenase encoded by *ALDH4A1*. PNPO disorders are secondary to the deficiency of the enzyme pyridox (am) ine 5′‐phosphate oxidase which converts both pyridoxine phosphate and pyridoxamine phosphate to PLP. Severe infantile hypophosphatasia is caused by reduced activity of TNSALP.^6^

A relatively new subgroup of pyridoxal phosphate binding protein (PLPBP) has recently been identified, previously known as proline synthetase cotranscribed (PROSC).^7^, ^8^, ^9^ Many variants in *PLPBP* gene seem to disrupt PLP homeostasis, which would explain the clinical presentation of PDE.^10^ Several biochemical abnormalities are characteristic for the each of PDE and can serve as diagnostic markers, but low PLP concentrations is a valid biomarker in the pretreatment cerebrospinal fluid in PLPBP patients.^4^

We present here clinical and molecular data of five cases in a French‐Canadian population, in which the same homozygous variants in *PLPBP* were identified.

## 2 MATERIALS AND METHODS

### 2.1 Recruitment of families and ethic statement

Persons affected with rare neuromuscular, neurodegenerative, metabolic, or poly‐malformative syndrome have been recruited in an interdisciplinary research program designated “Programme de Recherche et Innovation Sur les Maladies rarES” (PRISMES) at the CHU de Québec ‐ Université Laval (CHUQC‐UL) Research Centre.

### 2.2 DNA extraction and cell line immortalization

Blood samples were drawn from the recruited individuals. DNA was extracted using QIAamp DNA Blood kit (Qiagen, Toronto, Canada) according to the manufacturer's instructions.

### 2.3 Library preparation and whole exome sequencing

Libraries have been prepared from 3 μg of high‐quality genomic DNA using SureSelect XT human All exon V6+UTR kit (Agilent Technologies, Santa Clara, California).

### 2.4 Bioinformatics analyses of exome data and variant filtering

Data were processed using a pipeline adjusted from GATK Best Practices and the Snakemake workflow (Snakemake).^11^ Variants were functionally annotated based on data from SiFT,^12^ CADD,^13^ avsnp, Kaviar, ExAC, esp6500siv, 1000genomes, and Polyphen 2^14^ using Annovar and VEP^15^, ^16^ and the SEQR platform (SEQR).^17^

### 2.5 Haplotype analyses

Haplotype analysis was performed for the nuclear families including 17 informative single nucleotide polymorphisms 100 Kb upstream and 700 Kb downstream from the mutation site.

### 2.6 Western blot

Thirty‐five micrograms of lysate protein were subjected to sodium dodecyl sulfate‐polyacrylamide gel electrophoresis using 4% to 15% precast polyacrylamide gel (Bio Rad) followed by blot incubation with primary PROSC antibody (Sigma HPA023646).

Detailed information is given in Supporting Information 5.

## 3 RESULTS

In three families recruited in the project, we have identified in the affected probands the homozygous pathogenic mutation NM_001349346.2: c.370_373del, NP_001336275.1: p.Asp124fs (rs755595256) in *PLPBP* gene confirming vitamin B6‐dependent early‐onset epilepsy (OMIM 617290).

### 3.1 Clinical description (complete information in Supporting Information 1)

*Patient A* was born full term. Both parents were nonrelated French Canadians, from the Saguenay‐Lac‐St‐Jean region (see “PEDIGREE Patient A” in Supporting Information 2). The patient experienced tremors at the first few hours of his life and was transferred at a few hours of life to a tertiary center. A metabolic acidosis was discovered with a lactic acid level at 4.9 (mmol/L). Tonic clonic movements of the upper extremities and positive nystagmus were also noted, correlating with an abnormal electroencephalogram (EEG) upon arrival, revealing burst suppression activity and slow dysrhythmias as well as abnormal epileptic activity in the right temporal region. A cerebral magnetic resonance imaging (MRI) done at 2 days of life was normal. Phenobarbital was initially started, then replaced by Levetiracetam as well as Thiamine, Biotine, Riboflavin, Pyridoxal 5‐phosphate, Folic acid, and Coenzyme Q10, leading to improvement in condition. The second hospitalization was required after months due to refractory convulsions. In the context of a recent interruption of pyridoxal‐5′‐phosphate, he developed epileptic spasms prompting to start Vigabatrin. EEG showed modified hypsarrhythmia that improved after reintroduction of pyridoxal‐5′‐phophate. A global developmental delay as well as axial hypotonia was noted.

**FIGURE 2:**
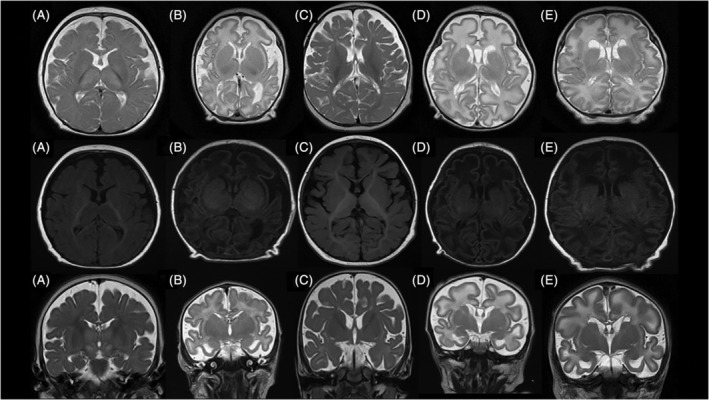
Brain MRI features. First row: axial T2 sequence; second row: axial T1 sequence; third‐row: coronal T2 sequence. A, Patient A at 2 months almost normal with mild delayed myelination. Increased subarachnoid spaces. B, Patient B at day 3 delayed myelination with temporal cysts and broad gyration with increased subarachnoid spaces and cortico‐subcortical atrophy. C, Patient C at 10 months mild delayed myelination, increased subarachnoid spaces, and cortico/subcortical atrophy. D, patient D at 1‐month broad gyration, delayed myelination, temporal cysts increased subarachnoid spaces, and cerebral atrophy. E: patient E at day 3 broad gyration, delayed myelination, temporal cysts, increased subarachnoid spaces, and cortico‐subcortical atrophy

MRI at 2 months showed delayed myelination (Figure 2) but a repeated brain MRI at the age of 6 months while on Vigabatrin, was suggestive of a possible metabolic anomaly on the spectrum of findings correlating with Leigh's disease, prompting the investigation of a possible mitochondrial disorder, but we concluded it was secondary to Vigabatrin exposure. Muscle biopsy and specific mitochondrial panels were all negative.

Language development was strongly limited at 2 years, he used pictograms to communicate. He was recently diagnosed with severe autism spectrum disorder. He has microcephaly with a head circumference of 42 cm at 2 years. Current pyridoxine dosage is 600 mg a day and P5P 100 mg a day (Table 1), he has a seizure free period, and antiepileptic medications were slowly weaned off at age of two.

**TABLE 1:** Clinical symptoms

Observation	Patient A	Patient B	Patient C	Patient D	Patient E
DOB	January 23, 2017	October 1, 2017	September 22, 2012	September 7, 2016	August 1, 2014
Date of death	n/a	October 14, 2017	February 7, 2014	n/a	August 20, 2014
Birth weight (g)	3205	3110	3752	2790	3190
Head circumference (cm)	33	31,5	33	33,5	34
Perinatal anomalies	Meconium in amniotic fluid	Meconium in amniotic fluid Oligohydramnios	Emergency c‐section due to abnormal fetal heart tracing	Emergency c‐section due to abnormal fetal heart tracing Oligohydramnios	Meconium in amniotic fluid
Neonatal metabolic lactic acidosis (mmol/L)	↑4.9	↑5.0	↑6.0	↑12.3	↑8.5
APGAR	6‐9‐10	9‐10	8‐9	9‐10	9‐9
Age seizure onset	Day 0	Day 0	Day 1	Day 0	Day 0
EEG at onset	Abnormal	Abnormal	Abnormal	Abnormal	Abnormal
Type of seizure	Tonic‐clonic, upper extremities, Nystagmus +	Tonic‐clonic, upper and lower extremities Recurrent hiccups	Arms flexion movements	Tonic movements, Tremulations, Abnormal mouth movements	Abnormal eyes movements Extension clonic movements Spasms
Initial anticonvulsants	Phenobarbital Levetiracetam	Phenobarbital Levetiracetam Lorazepam	Phenobarbital Midazolam Lamotrigine	Phenobarbital Levetiracetam Lorazepam	Phenobarbital Levetiracetam Lorazepam Phenytoine
Age—first dosage B6	Day 1 of life	Day 1 of life	Day 8 of life	Day 3 of life	Day 1 of life
Dosage, admin first dose	P5P 50 mg TID	P5P 30 mg TID	P5P 25 mg TID	Pyridoxine 85 mg DIE × 7 d Pyridoxine 42.5 mg BID	P5P 50 mg initial dose P5P 25 mg BID
Withdrawal trial of B6?	Yes, at 2 mo.	No, suspended at day 4 of life	Yes, at 6 wk of life	Yes, at day 29 of life and 1.5 y	Yes, at 3 d
*Result if yes*	Increased refractory convulsions	n/a	Increased convulsions	Recurrence of convulsions	n/a
MRI (Figure 2)	N on day 2 AB at 6 mo.	AB on day 1 AB on day 4	AB on day 4 AB at 2 mo. AB at 6 mo.	AB on day 1 AB on day 8	AB on day 3 AB on day 11
Latest anticonvulsant regimen	Wean off Nitrazepam Phenobarbital	n/a	Clonazepam Valproic acid Lacosamide Vigabatrine and Topiramate	Wean off Levetiracetam Topiramate Clobazam	Phenobarbital
Latest B6 dose	P5P 100 mg q4h	n/a	P5P 25 mg TID	P5P 75 mg QID Pyridoxine 42.5 mg BID	P5P 25 mg BID
Evolution of epilepsy	Stabilization	Deceased	Deceased	Stabilization	Deceased
Last EEG (2 y old)	Activity linked to complicated breath holding spells Absence of definite epileptic activity	n/a	n/a	Paroxystic and intermittent slow wave activity Absence of clear epileptiform elements	n/a
CSF					
Neurotransmitter metabolites	N	*↓ 5‐hydroxyindoleacetic acid*	N	N	No data
Alpha aminoadipic semialdehyde	N	N	n/a	n/a	No data
4 hydroxybutyric	N	N	n/a	n/a	No data
GABA	n/a	n/a	N	N	No data
Specific biom. for pyridoxine resp. seizures	Absent	Absent	n/a	n/a	No data
Pyridoxal 5′‐phosphate	N on day 2 N at 1 y	N on day 4	n/a	n/a	No data
Pyruvic acid (60‐190)	n/a	↓25	N	↓27	N
Plasma					
Acylcarnitines	N	N	N	N	N
AA GLycine level	N	N	N	N	N
Acide pyruvique (<80 μmol/L)	↑230	↑249	↑110	↑390	↑129
Creatine kinase (50‐200 U/L)	↑5629	↑2486	↑4405	↑6706	↑8238
Urine					
AA	N	N	n/a	N	N
Development					
Speech	Absent at 2 y	n/a	Not acquired at death	Repeats words at 3 y. No sentences	n/a
Sitting months	12	n/a	Not acquired at death	12	n/a
Walking months	24	n/a	Not acquired at death	23	n/a
Fine motor function	n/a	n/a	Not acquired at death	Delayed	n/a
Weight percentile	50th	n/a	50th	50th	n/a
Height percentile	25 mo.: 15th 31 mo.: 25th	n/a	n/a	14 mo.: 15th 38 mo.: <0.1	n/a
Microcephaly	No	Reduced CP at birth	Yes progressive	Yes—progressive <2nd percentile	No

Abbreviations: ↑, increase; ↓, decrease; AA, amino acids; AB, abnormal; CP, head circumference; CSF, cerebrospinal fluid; EEG, electroencephalogram; GABA, gamma aminobutyric acid; MRI, magnetic resonance imaging; N, normal; n/a, not applicable.

*Patient B* was born at 37 weeks. Within the first few hours of his life, patient presented respiratory distress, tremor, erratic movements of limbs, and lower limb rigidity. Metabolic acidosis was discovered with an elevated lactic acid level up to 5.0 mmol/L. An initial EEG revealed abnormal activity with burst suppression pattern. An MRI showed diffuse cerebral atrophy and delayed cerebral maturation and myelination (Figure 2). He initially received Phenobarbital, Levetiracetam and Lorazepam, vitamin treatment was initiated on day 1 of life and consisted of Biotin, Thiamine, Riboflavin, B12, Coenzyme Q10, and Pyridoxal 5′‐phosphate (P5P) 90 mg a day. At day 4, treatments including P5P were suspended in order to prioritize comfort.

The patient had older brother (patient C) who died of a severe epileptic encephalopathy (see “PEDIGREE Patient B and C” in Supporting Information 2 ). Both parents were originally from the Saguenay‐Lac‐St‐Jean region, with no notion of consanguinity. He received P5P 75 mg a day since eighth day of life (Table 1).

*Patient C* was born at term and developed refractory seizure at 24 hours of life. EEG demonstrated burst suppression pattern. He had multiple types of seizures with predominated tonic seizures. The condition worsened over a few months, patient showed severe global developmental delay and severe refractory seizures. He died at the age of 16 months from withdraw of care. His MRI at 10 months showed cortical/subcortical atrophy with mild delayed myelination but normal spectroscopy (Figure 2). Postmortem autopsy did not have any conclusions about myelination.

*Patient D* was born prematurely at 36 weeks, with a normal birth weight for gestational age. Within the first few hours of life, patient presented respiratory distress followed by stereotypical tonic movements associated with desaturation and tremor necessitating a transfer to a tertiary center. Lab results revealed a severe lactic acidosis with levels at 12.3 mmol/L.

The patient had an older brother (patient E) who died, his pathology reported moderate microcephaly and hypoxic ischemic changes (see “PEDIGREE Patient D and E” in Supporting Information 2). Both parents were originally from the Saguenay‐Lac‐St‐Jean region, with no notion of consanguinity.

An initial EEG revealed a low voltage rhythm and a highly abnormal tracing indicative of a severe bihemispheric encephalopathy with suppression of electrogenesis. A cerebral MRI at day 1 of life showed a diffuse anomaly on the white matter, extending toward subcortical regions, suggestive of metabolic anomalies. A second MRI at 1 month of age showed persistence of delayed myelination in both hemispheres and the cerebellum as well as multiples subcortical cysts (Figure 2).

Patient initially received Phenobarbital, Levetiracetam, Lorazepam, Sodium dichloroacetate, Thiamine, Carnitine, Coenzyme Q10, Vitamin B12, and Biotin. Pyridoxine and Folinic acid were added initially on the third day until 29th day when they were suspended. The initial dosage of Pyridoxine was 85 mg a day.

The patient presented a global developmental delay. He was able to walk independently at 23 months. At the age of 3 years, he lacked an expressive language. Social contact was reported as present. At the age of 27 months, his head circumference was 46.2 cm (second percentile), indicative of a progressive microcephaly. Antiepileptic medications were decreased after the second year of life without any other seizure episode. The latest treatment regimen is P5P 300 mg and Pyridoxine 85 mg a day.

*Patient E* was born at term. He developed severe refractory seizures within the first 4 hours of life. EEG demonstrated severe burst suppression pattern. His condition did not improve despite loading doses of vitamins. Withdraw of care was decided by parents according of results of brain MRI at day 3 of age showing diffuse white matter abnormalities associated with broad gyration and cortico/subcortical atrophy and poor neurological outcomes. Loading dose was P5P 50 mg a day.

### 3.2 Genetic finding

Whole exome sequencing of the probands identified homozygous pathogenic deletion NM_001349346.2: c.370_373del, NP_001336275.1:p.Asp124fs (rs755595256) in *PLPBP* gene. Segregation analysis confirmed heterozygous carrier status in all parents. Allelic frequency of this mutation in gnomAD database is 0.00007. It is mostly observed in the North Western European populations and completely absent from Latinos, Ashkenazi Jewish, East Asian, and Finnish populations (gnomAD). The CADD, PROVEAN, and Polyphen annotations predicted a damaging frameshift alteration. According to ORF (open reading frame) finder tools, the deletion in the exon 5 of *PLPBP* gene leads to a truncated protein of 124 amino acid instead of 285. Mutation c.370_373del, NP_001336275.1:p.Asp124fs was previously described.^18^

Haplotype analysis of 100 to 700 Kb genomic region upstream and downstream of the deletion showed unique haplotype between three of the affected and nonaffected members of the three families. This leads to the hypothesis about founder effect of the mutation of French Canadians in Saguenay‐Lac‐St‐Jean region (see Supporting Information 3).

Interestingly, all affected probands also shared one single mitochondrial haplotype T2b3 and two rare variations in the mitochondrial genome m.801A>G and m.5166A>G. This in addition to genome analysis of other patients that are part of our PRISMES project suggests that those mitochondrial variations appear to be uniquely associated with the described mutation in the *PLPBP* gene (data not shown).

### 3.3 Functional impact of the mutation

As predicted by the in silico tools, Western blot analysis confirmed complete absence of the PLPBP protein in the lysate extracted from the immortalized B cells of the patient A and C, PLPBP protein was present in the protein extract of all patients' parents (Supporting Information 4).

## 4 DISCUSSION

Founder effect in French Canadian population, particularly from Saguenay‐Lac‐St‐Jean region, is an essential reason of recurrent neurogenetic autosomal recessive disorders. The genetic basis for some of them are well known and there is an effective carrier screening for six genetic neurodegenerative disorders in that region, but we can assume that other recessive disorders could be highly represented in this population as well.

Our research project identified three families with probands affected by vitamin B6‐dependent epilepsy having homozygous pathogenic mutation c.370_373del, p.Asp124fs in *PLPBP* gene.

Haplotype analysis of the three affected probands in genomic region proximal to the mutation, permitted us to identify single shared haplotype. Family tree analysis did not allow us to find a single common ancestor and all families denied any consanguinity. All families are originally from the Saguenay‐Lac‐St‐Jean region of Quebec province in Canada. Saguenay‐Lac‐St‐Jean historically is the relatively isolated region founded by mostly French emigrants during the 17th century, nine generations ago confirmed by recent population studies data.^19^ Presence of this rare variation in the persons of North‐Western European origin in the gnomAD database let us to speculate that mutation c.370_373del, p.Asp124fs carrier has been among the founders of the present French Canadian population, and due to the population expansion with limited gene pool, the variant become frequent in the persons of Saguenay‐Lac‐St‐Jean origin.

Clinical presentation of PDE strongly mimics mitochondrial disorders, and it was reported as a strong pitfall of ability to easy diagnose PDE.^20^ All our patients presented with metabolic acidosis, they had severe lactic and pyruvate acidosis, normal metabolic profile, and a high level of creatine kinase. It was a constellation of clinical and biochemical findings characteristic for some rare mitochondrial pathologies, like Saguenay‐Lac‐Saint‐Jean founder mutation in *LRPPRC* gene, or mutations in *DNM1L* or *SLC25A22* genes.^21^, ^22^ This directed to the assumption of mitochondrial pathology and initial treatment with “mitochondrial cocktail,” which included pyridoxine, but a lower dosage as necessary for PDE.

Our patients were as well tested for the mitochondrial disorders and mitochondrial genome sequencing which revealed for all of them two variations with unknown clinical significance (m.801A>G and m.5166A>G). The variant m.801A>G in *MT‐RNR1* gene is rare, but it has been published as deaf‐associated mutation and it is registered in MITOMAP in six individuals representing different U, D, H, L, and M haplogroups.^23^ The second variant m.5166A>G is a rare nonsynonymous mutation of *MT‐ND2* gene published only once, and not related to any clinical symptoms.^24^ It is difficult to speculate causality for those mutations and epistatic effects without more information supporting this line of inquiry. However, association with above‐mentioned founder effect could indicate that a single individual female introduced *PLPBP* mutation c.370_373del, p.Asp124fs in Quebec.

Previously described affected persons by mutation c.370_373del, p.Asp124fs in *PLPBP* gene came from the Canada's Cree First Nation and the United States' African American consanguineous families as explained previously.^18^ PDE caused death for one of the patients at 8 weeks, she also had a cystic malformation in her brain MRI, and the second patient had only partial response to P5P treatment of 40 mg/(kg d).

Natural history of the disease in the homozygous persons even from the one family is not uniform in the present study. The seizure spectrum is wide in these patients, almost all types of neonatal seizures could present, but mainly tonic seizures are the first observed. A cerebrospinal fluid (CSF) analysis was done in four of the patients and revealed normal neurotransmitter metabolite levels as well as P5P. We suggest that genetic testing should be the initial investigation in patients with neonatal refractory seizures. Treatment with pyridoxine and or P5P has to be started immediately and continued until the results of genetic analysis received.

Of five patients identified in the project, only two are currently alive, but they suffer from the severe developmental delay and autism spectrum disorder. However, we identified some important factors that might explain this clinical variability. The mothers of two alive patients took high doses of pyridoxine during pregnancy as antiemetic drug doxylamine succinate‐pyridoxine HCl. The fact that only two patients survived demonstrates the severity of this mutation. Also, it raises the question of antenatal pyridoxine supplement that could bring a better outcome for the milder phenotypes (missense mutations). Second, patients received specific treatment early and were maintained on pyridoxine even without confirmatory genetic result. Both patients despite refractory epilepsy at beginning with vitamin supplements showed a significant decrease in seizure activity up to the possibility to remove from the anticonvulsive therapy, while keeping them only on vitamins regimen. Deceased patients initially received a lower dosage of pyridoxine due to the working hypothesis of mitochondrial disease.

We could not clearly explain this phenomenon except if the part of pyridoxine and the gamma aminobutyric acid (GABA)‐related pathway is less predominant after 2 years. All patients presented with mild to moderate cerebral atrophy on brain MRI and clinically with a progressive microcephaly, those findings were confirmed in autopsy (patient C). We might hypothesize that pyridoxine and pyridoxal pathway play a very important role in prenatal and neonatal brain maturation and connectivity explaining the poor neurodevelopmental outcome despite early treatment. Further studies of these patients implying white matter function could bring new highlights in the B6 networking.

As previously mentioned, the mutation c.370_373del, p.Asp124fs causes severe disease phenotype with early delayed brain myelination and cortical/subcortical brain atrophy. The most noteworthy finding in the brain MRI is the same pattern of delayed myelination with a cyst formation in the frontal and temporal lobes that can be used as a marker of the disease. Myelination improves after months or years with treatment.

This study extends our understanding of vitamin B6‐dependent early‐onset epilepsy with founder mutation in French Canadian population. Further studies are needed to alleviate the neurodevelopmental burden in these patients.

## CONFLICT OF INTEREST

The authors declare no potential conflict of interest.

## AUTHOR CONTRIBUTIONS

Maitou Pal: conception and design, analysis and interpretation of data, drafting the article or revising it critically for important intellectual content, biochemical pathway analysis; Baiba Lace: conception and design, analysis and interpretation of data, drafting the article or revising it critically for important intellectual content, haplotype analysis, mitochondrial data analysis; Yvan Labrie: conception and design, analysis and interpretation of data, drafting the article or revising it critically for important intellectual content, molecular biology, bioinformatic analysis; Nathalie Laflamme: conception and design, analysis and interpretation of data, drafting the article or revising it critically for important intellectual content, molecular biology; Nadie Rioux: conception and design, analysis and interpretation of data, drafting the article or revising it critically for important intellectual content, patient recruitment; Samarth Thonta Setty: conception and design, analysis and interpretation of data, drafting the article or revising it critically for important intellectual content, bioinformatic analysis; Marc‐Andre Dugas: conception and design, analysis and interpretation of data, drafting the article or revising it critically for important intellectual content, concept of project, patient recruitment; Louise Gosselin: conception and design, analysis and interpretation of data, drafting the article or revising it critically for important intellectual content, patient recruitment; Arnaud Droit: conception and design, analysis and interpretation of data, drafting the article or revising it critically for important intellectual content, bioinformatic analysis; Nicolas Chrestian: conception and design, analysis and interpretation of data, drafting the article or revising it critically for important intellectual content, neuro follow‐up, MRI interpretation; Serge Rivest: conception and design, analysis and interpretation of data, drafting the article or revising it critically for important intellectual content, concept of project.

## ETHICS STATEMENT

All procedures followed were in accordance with the ethical standards of the responsible committee on human experimentation (institutional and national) and with the Helsinki Declaration of 1975, as revised in 2000 (World Medical Association *Declaration of Helsinki*: ethical principles for medical research involving human subjects. World Medical Association. JAMA. 2013 Nov 27;310(20):2191‐2194. doi: 10.1001/jama.2013.281053. Research ethics board approval of the study design was obtained from the Comité d’éthique de la recherche (CER) du CHUQC‐UL.

## PATIENT CONSENT

All participants and/or their parents provided written informed consent prior to enrolment.

## Supporting information


**Supplement 1**.

**Supplement 2**.

**Supplement 3**.

**Supplement 4**.

**Supplement 5**.
