# Supplementary material for: A founder mutation in the PLPBP gene in families from Saguenay‐Lac‐St‐Jean region affected by a pyridoxine‐dependent epilepsy
Source: JIMD Rep. 2021 Feb 23;59(1):32–41. doi: 10.1002/jmd2.12196 (PMC8100403; doi:10.1002/jmd2.12196)
Supplement: Supplementary file 1 — Supplement 1. [file JMD2-59-32-s001.pdf]

Supplement 1

Patient A (2017/01/23)

Patient A was born full term. Obstetrical history was nonsignificant except for gestational diabetes controlled with diet.

At a few hours of life tonic clonic movements of the upper extremities, nystagmus and an abnormal EEG were noted. EEG showed "burst suppression" activity and slow dysrhythmias as well as abnormal epileptic activity in the right temporal region. A cerebral MRI was done at the second day and was normal. Phenobarbital was initially started, then replaced by Levetiracetam as well as Thiamine, Biotine, Riboflavin, Pyridoxal 5-phosphate, Folic acid and Coenzyme Q10. Biochemical investigations shown normal plasma and urinary amino acid and organic acid levels. The patient's CGH and karyotype were normal as well.

A CSF analysis revealed normal neurotransmitter metabolite levels as well as more specifically normal alpha aminoadipic semialdehyde and 4-hydroxybutyric acid levels. Specific biomarkers for pyridoxine responsive seizures that would've been noted on the metabolite chromatogram were absent. CSF Pyridoxal-5 phosphate levels were consistently normal at day two of life and at a year.

A second hospitalization was required at two months of life for refractory convulsions, in the context of a recent interruption of Pyridoxal 5-phosphate treatment. The former was reinitiated along with the introduction of Vigabatrin, an increase in dose of Levetiracetam and a temporary regular dose of Prednisone. The patient had a few relapses during withdrawal trials of Sabril and anticonvulsant dosage adjustments (Topiramate, Vigabatrin, Levetiracetam). A strong response to Vigabatrin as well as a partial response and dependency to Pyridoxine were noted as per evolution.

Symptoms were ultimately controlled with his last EEG at the age of two revealing activity linked to complicated breath-holding spells but no definite epileptic activity. The patient's latest medication regimen consists of Nitrazepam 0.8 ml TID, Phenobarbital 18 mg BID and Pyridoxal 5-phosphate 100 mg every four hours.

A global developmental delay as well as axial hypotonia was noted. Patient started sitting independently at the age of one, being able to stand with help at the age of 15 months, walking at the age of two. Language development is limited with an inability to talk at two years old but uses pictograms to communicate. Visual contact is present but limited. An autism spectrum disorder is currently being investigated. He maintained a weight in the 50th percentile, height between the 3rd and 15th percentile.

Patient B (DOB:2017/10/01)

Patient B was born at 37 weeks, with a normal birth weight and reduced head circumference (between the 3rd and 10th percentile).

Prenatal history was nonsignificant, with a premature rupture of membranes and subsequent Caesarean section due to the refusal of a VBAC, without any major complications (APGAR 9–10, stained amniotic liquid, oligohydramnios).

The patient had relevant family history having had an older brother who died at the age of 16 months in the context of a severe epileptic encephalopathy. An extensive genetic investigation was undertaken at the time (CSF neurotransmitter metabolites, metabolic screening, mitochondrial disease investigations) leading to no conclusive results. He had another elder

brother in good health. Both parents were originally from the Saguenay-Lac-St-Jean region, with no notion of consanguinity.

Within the first few hours of life, patient presented respiratory distress, hypopnea, tremor, erratic movements of superior and inferior limbs and recurrent hiccups as well as episodes of lower limb rigidity. Upon investigation, metabolic acidosis was discovered with an elevated lactic acid level going until 5.0 (mmol/L). An initial EEG at day 0 of life revealed very abnormal activity with severe suppression of cerebral activity, numerous muscular artefacts and reduced the voltage of the base rhythm. An EEG was repeated on day four, revealing severe burst suppression pattern.

An MRI showed diffuse cerebral atrophy and delayed cerebral maturation and myelination at the day one.

He initially received Phenobarbital, Levetiracetam and Lorazepam. Vitamin treatment was initiated on day 1 of life and consisted of Biotin 10 bid, Thiamine 50 mg IV bid, Riboflavin 25 die, B12 1 mg IM die, Coenzyme Q10 and Pyridoxal 5-phosphate (P5P) 30 mg/kg/die.

At day 4 of life, due to the severity of the situation, lack of significant improvement and no progression witnessed by the family, it was decided that a palliative care approach would be most appropriate. Treatments including P5P were suspended in order to prioritize comfort, patient was treated with Lorazepam, Opioids and Anticonvulsants as per needed.

Biochemical investigations were relatively normal with slight nonsignificant and non-specific variation in plasmatic amino acid levels. Amino acid CSF levels were also considered normal. CSF neurotransmitter metabolites were measured, revealing a low level of 5-hydroxyindoleacetic acid, potentially suggestive of an anomaly regarding serotonin metabolism. CSF levels of P5P, Alpha aminoadipic semialdehyde and 4-hydroxybutyric acid were all within reference ranges. Patient's karyotype was normal. CGH revealed a gain of 52 oligonucleotides on chromosome 8. This same anomaly was found when the father was tested, rendering it is a benign copy number variation.

The patient had deceased at 14 days of life. A post-mortem autopsy was conducted on the brain, revealing cortical developmental immaturity and numerous hypoxic zones.

<u>Patient D (DOB: 2016/09/07)</u>

Patient D was born prematurely at 36 weeks and 4 days, with a normal anthropometric measurement for the gestational age. Obstetrical history was nonsignificant except for gestational diabetes controlled with diet. An emergency Caesarean section was required due to atypical foetal heart tracing. APGAR at birth was evaluated at 9–10.

Within the first few hours of life, patient presented respiratory distress followed by stereotypical tonic movements associated with desaturation, tremor and repetitive mouth movements, necessitating a transfer to a tertiary centre. Lab results revealed a severe lactic and pyruvic acidosis with levels at 12.3 (mmol/L) and 390 (μmol/L) respectively.

Both parents were originally from the Saguenay-Lac-St-Jean region, with no notion of consanguinity between families.

An initial EEG at day 0 of life revealed a low voltage rhythm, intermittent theta waves and a highly abnormal tracing indicative of a severe bihemispheric encephalopathy with suppression of electrogenesis. A cerebral MRI at day one of life showed a diffuse abnormality on

the white matter, extending toward sub-cortical regions, suggestive of metabolic anomalies. A second MRI at day eight of life showed persistence of white matter signal anomalies in both hemispheres and the cerebellum as well as the apparition of multiples subcortical cysts.

Patient initially received Phenobarbital, Levetiracetam and Lorazepam as per needed as well as Sodium Dichloroacetate.

On day 1 of life, he was administered Thiamine 25 mg IV BID, Carnitine, Coenzyme Q10, Vitamine B12, and Biotin. Pyridoxine at 85 mg PO die and Folinic acid at 10 mg PO die were added initially for seven days on day 3 of life and were then pursued further at 42.5 mg twice daily and 10mg once daily respectively, until day 29 of life, when they were suspended. State was relatively controlled upon discharge at the age of one month at nine days. Medication at discharge consisted of Levetiracetam, Topiramate, Carnitine, Coenzyme Q10, Thiamine, Riboflavin and Vitamin D.

Patient was readmitted after two days due to the recurrent convulsions (associated to a potential context of dehydration) with an EEG showing multiple epileptiform dysrhythmic anomalies. Folinic acid and Pyridoxine (25 mg IV twice daily) were re-instated at an anticonvulsant doses were increased, leading to a relatively stable state.

An extensive biochemical investigation was undertaken. Plasma amino acid, acylcarnitine profiles, long chain and very long chain fatty acids were normal. Urinary amino acid, orotic acid and purine/pyrimidine levels were also within reference ranges. CSF neurotransmitter levels were relatively normal with an isolated relative decrease in homovanillic acid levels. Amino acid levels were difficult to evaluate due to the contamination by blood. CSF GABA levels were normal. Cytogenetic investigations revealed a normal CGH and karyotype. Several gene panels were conducted. An epilepsy panel (by Gene Dx, containing 87 genes) was negative. A combined nuclear mitochondrial and lactic acidosis-pyruvate panels was also negative as well as a "Vanishing white matter, dysmyelinating and hypomyelinating leukodystrophy panel" and "Aicardi Gouttières panel." A muscle biopsy was then done, revealing no anomalies.

Patient was hospitalized twice the following year for convulsions associated with an infectious or dehydrated condition. His last hospitalization was in the context of a withdrawal trial and decrease in his Pyridoxine dosage, with resolution of symptoms when a previous dose was reinitiated (42.5 mg twice daily).

His evolution has been relatively favourable. His latest medication regimen consisted of: Clobazam, Topiramate, Pyridoxal phosphate, Pyridoxine and Folinic acid. The most recent EEG on file was done at the age of two revealed paroxystic and intermittent slow wave activity and the absence of clear epileptiform elements.

The patient's last cerebral MRI was done at the age of 11 months, showing the progression of white matter atrophy with prominent ventricles as well as persistence in increased white matter signalling in bifrontal areas and the posterior portion of the pons.

The patient presented a global developmental delay. He was able to stand with help at 14 months and walk independently at 23 months. His fine motor skills were evaluated at a corresponding age of nine months at the age of three. At 2.5 years, he was able to make sounds but no clear words and had limited comprehension skills. At the age of three, he is able to repeat words but does not use them. Social contact was reported as present. At the age of 14 months, patient's weight was in the 50th percentile, height and cranial circumference was at the 15th percentile. Most recent measurements show a sharp drop in percentile regarding head

circumference which would correspond to a level inferior to the 2nd percentile, indicative of a progressive microcephaly.
