# Supplementary material for: A founder mutation in the PLPBP gene in families from Saguenay‐Lac‐St‐Jean region affected by a pyridoxine‐dependent epilepsy
Source: JIMD Rep. 2021 Feb 23;59(1):32–41. doi: 10.1002/jmd2.12196 (PMC8100403; doi:10.1002/jmd2.12196)

PEDIGREE Patient A

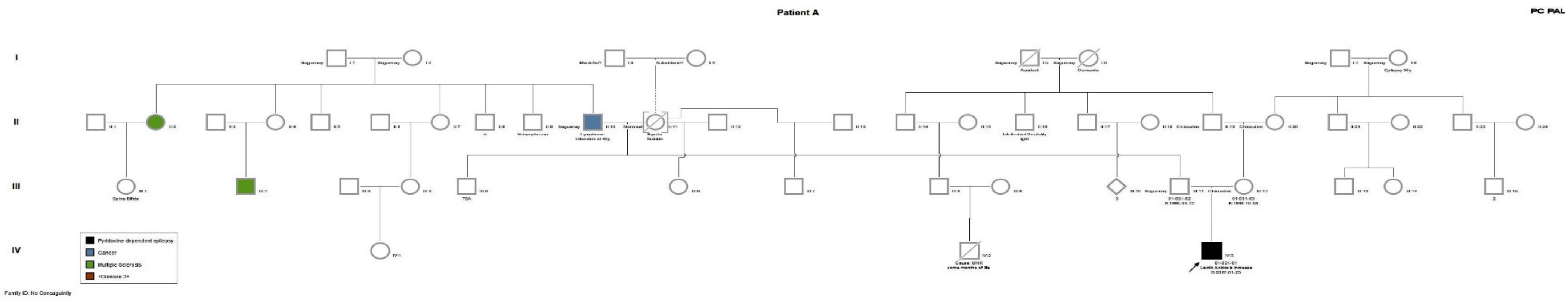

PEDIGREE Patient B and C

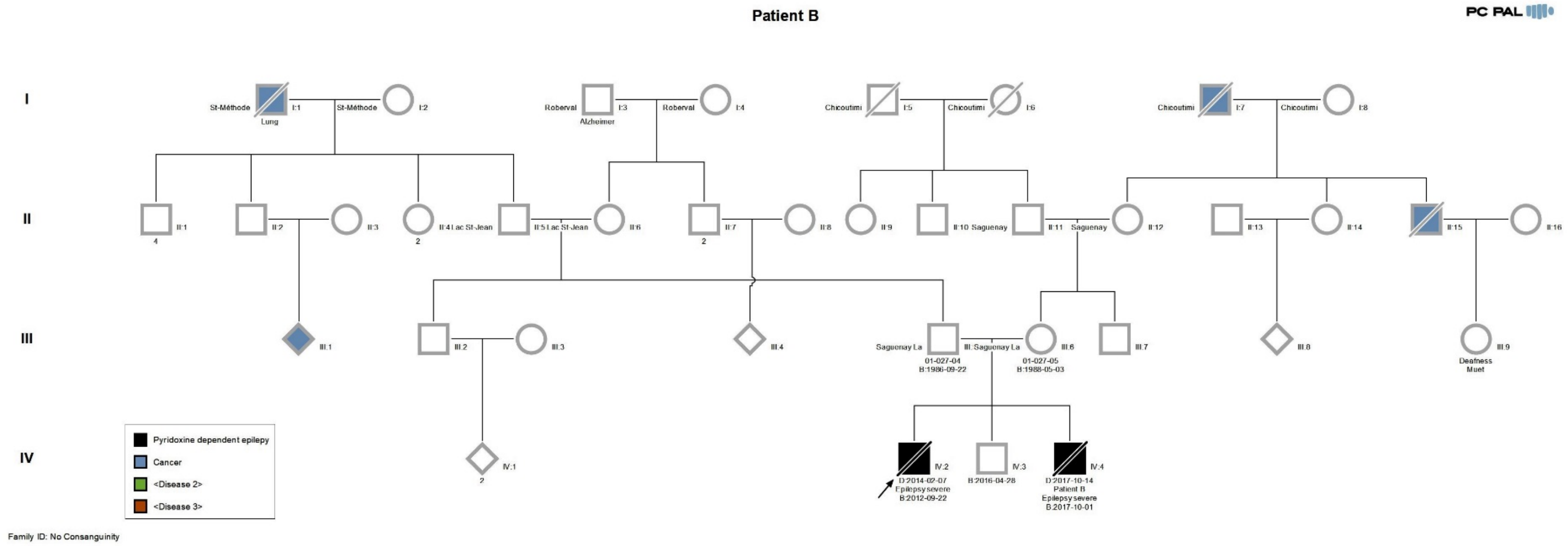

PEDIGREE Patient D and E

Patient D

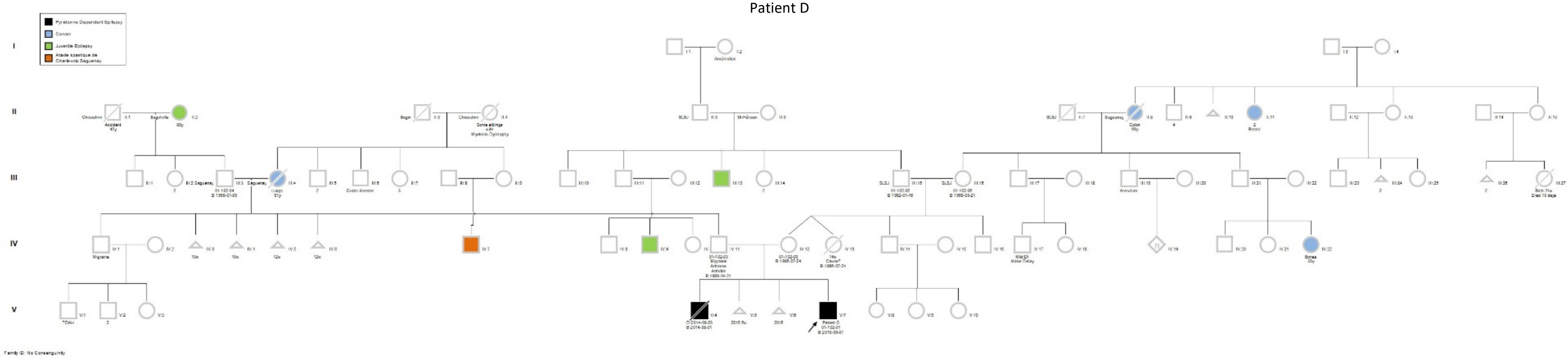

Supplement: Supplementary file 2 — Supplement 2. [file JMD2-59-32-s005.pdf]
