# Supplementary material for: A founder mutation in the PLPBP gene in families from Saguenay‐Lac‐St‐Jean region affected by a pyridoxine‐dependent epilepsy
Source: JIMD Rep. 2021 Feb 23;59(1):32–41. doi: 10.1002/jmd2.12196 (PMC8100403; doi:10.1002/jmd2.12196)
Supplement: Supplementary file 3 — Supplement 3. [file JMD2-59-32-s004.pdf]

Supplement 3.

Similar Haplotypes for the three families

homozygous recessives variants

		Patient A						Patient B						Patient D						
**Chr position**	**Ref**	**Father**		**Mother**		**Affected**		**Father**		**Mother**		**Affected**		**Father**		**Mother**		**Affected**		
36924917	A	G	G	G	G	G	G	G	G	G	G	G	G	G	G	G	G	G	G	intergenic
37374556	C	T	T	T	T	T	T	T	T	T	T	T	T	T	T	T	T	T	T	ncRNAintronic
37455059	G	G	A	A	A	A	A	A	A	G	A	A	A	A	A	A	A	A	A	intergenic
37455520	C	C	A	A	A	A	A	A	A	C	A	A	A	A	A	A	A	A	A	intergenic
37455990	G	G	C	C	C	C	C	C	C	G	C	C	C	C	C	C	C	C	C	intergenic
37456438	A	A	G	G	G	G	G	G	G	A	G	G	G	G	G	G	G	G	G	intergenic
37592257	G	C	C	C	C	C	C	C	C	C	C	C	C	C	C	C	C	C	C	doenstream
37613910	G	G	A	G	A	A	A	G	A	G	A	A	A	G	A	G	A	A	A	UTR3
37614504	G	G	A	G	A	A	A	G	A	G	A	A	A	G	A	G	A	A	A	UTR3
37620076	A	G	G	G	G	G	G	G	G	G	G	G	G	G	G	G	G	G	G	exonic
37630321*	***CAGA***	***CAGA***	-	***CAGA***	-	-	-	***CAGA***	-	***CAGA***	-	-	-	***CAGA***	-	***CAGA***	-	-	-	***exonic***
37686749	A	A	G	G	G	G	G	G	G	G	G	G	G	A	G	G	G	G	G	intronic
37688572	A	A	C	A	C	C	C	C	C	C	C	C	C	A	C	C	C	C	C	intronic
37699195	C	C	G	C	G	G	G	G	G	G	G	G	G	C	G	G	G	G	G	Exonic
37728017	A	A	G	A	G	G	G	A	G	G	G	G	G	G	G	G	G	G	G	Exonic
37728019	T	T	G	T	G	G	G	T	G	G	G	G	G	G	G	G	G	G	G	Exonic
37730368	G	G	A	G	A	A	A	G	A	A	A	A	A	A	A	A	A	A	A	Exonic

*Mutation C368-371del PLPBP **

Transmited allele from mother

Transmitted allele from father
