# Supplementary material for: A founder mutation in the PLPBP gene in families from Saguenay‐Lac‐St‐Jean region affected by a pyridoxine‐dependent epilepsy
Source: JIMD Rep. 2021 Feb 23;59(1):32–41. doi: 10.1002/jmd2.12196 (PMC8100403; doi:10.1002/jmd2.12196)
Supplement: Supplementary file 5 — Supplement 5. [file JMD2-59-32-s002.docx]

**Methods**

**Recruitment of families and Ethic statement**

Persons affected with rare neuromuscular, neurodegenerative, metabolic or poly-malformative syndrome have been recruited in an interdisciplinary research program designated "Programme de Recherche et Innovation Sur les Maladies rarES" (PRISMES) at the CHU de Québec - Université Laval (CHUQC-UL) Research Centre. PRISMES aims to recruit patients and their family members as a trio or more.

Research ethics board approval of the study design was obtained from the Comité d’éthique à la recherché (CER) du CHUQC-UL. All participants and/or their parents provided written informed consent prior to enrolment.

**DNA extraction and cell line immortalization**

Peripheral blood mononuclear cells were isolated using Ficoll gradient density method. Blood samples were slowly deposed on a Ficoll plaque gradient (GE Healthcare) and centrifuged 45 min at 1800 RPM. The central cloudy layer was washed and infected with titrated Epstein-Barr Virus. Infected cells were grown and transferred in culture flask. Clumps of immortalized B cells were then counted and cryopreserved.

**Library Preparation and Whole Exome Sequencing**

Libraries have been prepared from 3 µg of high-quality genomic DNA using SureSelect XT human All exon V6+UTR kit (Agilent Technologies, Santa Clara, USA). This technology is already automated and up-and-running at the CHUQC-UL Research Center. DNA was fragmented on a Covaris instrument (Covaris, Woburn, MA, USA) and adaptor-tagged to an average size of ~275-300bp. Libraries were then be subjected to exome capture. Three libraries with unique index were pooled together in equimolar ratio and sequenced at a mean coverage of 100X on an Illumina HiSeq2500 for paired-end 125 bp sequencing at both sites.

**Bioinformatics Analyses of Exome Data and Variant Filtering**

Data was processed using a pipeline adjusted from GATK Best Practices and the Snakemake workflow (Snakemake). Raw data was demultiplexed using Illumina’s proprietary bcl2fastq to get to an open format. Then raw reads were trimmed using Trimmomatic (Bolger et al. 2014) and mapped to human reference genome (hg19) using BWA (Li and Durbin 2010). Duplicated reads were flagged using Picard MarkDuplicates (Picard Toolkit, 2018) and base score recalibration was performed using GATK BaseRecalibrator (McKenna et al. 2010).

**Western blot**

Protein was extracted from a lysate of immortalized B cells and measured by the Quantipro BCA assay kit (Sigma-Aldrich). 35 ug of lysate protein were subjected to SDS-polyacrylamide gel electrophoresis using 4–15% precast polyacrylamide gel (Bio Rad) followed by blot incubation with primary PROSC antibody (Sigma HPA023646), diluted 1/1000 in 5% skim milk and 0.1 M TRIS-buffered saline-Tween X-100 (TBS-T). Primary antibodies were detected with horseradish peroxidase (HRP)-conjugated secondary antibody diluted 1/5000 in 5% skim milk/TBS-T and revealed by enhanced chemiluminescence plus solution. Membrane was re-incubated with beta actin diluted 1/9000 as a positive control (MAB1501 Millipore).

References

1. Bolger AM, Lohse M, Usadel B (2014) Trimmomatic: a flexible trimmer for Illumina sequence data. Bioinformatics 30:2114–20. https://doi.org/10.1093/bioinformatics/btu170
2. Li H, Durbin R (2010) Fast and accurate long-read alignment with Burrows-Wheeler transform. Bioinformatics 26:589–95. https://doi.org/10.1093/bioinformatics/btp698
3. Picard Toolkit (2018) Broad Institute, GitHub Repository. http://broadinstitute.github.io/picard/; Broad Institute.
4. McKenna A, Hanna M, Banks E, et al (2010) The Genome Analysis Toolkit: a MapReduce framework for analyzing next-generation DNA sequencing data. Genome Res 20:1297–303. https://doi.org/10.1101/gr.107524.110
